# EFFECTIVENESS OF DIFFERENT INTERGENERATIONAL LEARNING PROGRAMS FOR ELDERS WITH MILD COGNITIVE IMPAIRMENT

**DOI:** 10.1093/geroni/igz038.422

**Published:** 2019-11-08

**Authors:** Li-Chan Lin

**Affiliations:** Institute of Clinical Nursing, National Yang-Ming University, Taipei, Taiwan

## Abstract

The purposes of this study was to investigate the efficacy of interpersonal relationships, cognition, instrumental activities of daily living, depression for elderly with cognitive impairment through intervention using the intergenerational somatosensory video game and company. The experimental design was used for this study. Eighty-nine elders with mild cognitive impairement and 180 adolescents were from nine junior and senior high school were recruited in this study. Eight day care centers were randomly assigned to the experimental group I (EGI) (5-week intergenerational somatosensory video game), the experimental group II (EGII) (8-week intergenerational somatosensory video game), the experimental group III (EGIII) (5-week intergenerational company), the experimental group IV (EGIV) (8-week intergenerational company) and control group for eight weeks of routine activities. Subjects were interviewed using structured instruments, including the Instrumental Activities of Daily Living (IADL), Token test, and Geriatric Depression Scale. Obtained data was analyzed using the Generalized Estimate Equation (GEE). The results revealed that there was no significant difference of eldsers’ characteristics among five groups. After intervention, instrumental activities of daily living scores in 8-week intergenerational somatosensory video game (EGII) and 8-week intergenerational company groups (EGIV) were significantly better than the control group. Token test score in 8-week intergenerational company (EGIV) were significant higher than that in the control group; while depression in 5-week intergenerational company (EGIII) was significantly lower thatn that in the control group. Based on the research findings, to arrange intergenerational action video game and intergenerational company to enhance elders’ attention, instrumental activities of daily living and decrease depression.

